# Representation and career trajectories of female principal investigators in clinical trials, 2010–2023

**DOI:** 10.1016/j.conctc.2025.101566

**Published:** 2025-10-30

**Authors:** Manuel Hermosilla

**Affiliations:** University of Illinois at Chicago, United States

## Abstract

**Background:**

Gender disparities persist in clinical trial leadership, with women underrepresented as principal investigators (PIs). While previous studies have documented these disparities, they have largely relied on limited datasets focused on specific therapeutic areas. Previous studies have also neglected potential gender differences in trialist career trajectories. This study examines gender representation in clinical trial leadership using the most comprehensive dataset available, assessing trends over time and gender differences in trialist career trajectories.

**Methods:**

I analyzed a large sample of clinical trials submitted to ClinicalTrials.gov from 2010 to 2023. PI gender was inferred using algorithmic classification based on first names. I estimated the probability of female PI leadership for each trial year using logistic regression, adjusting for trial characteristics. To assess career trajectories, I examined the number of trials led by male and female PIs within fixed time horizons and assessed gender differences and their evolution in time using Poisson regression models.

**Results:**

Among nearly 160,000 trials analyzed, female representation increased from 32 % in 2010 to 41 % in 2023. Female PI leadership remained lowest in cardiovascular trials (20 %) and highest in behavioral intervention trials (50 %). Although male PIs lead more trials than female PIs over all horizons after entering the field, the gap has narrowed over time. In 2010, male PIs led 7 % more trials than female PIs within their first three years after entering the field; by 2020, this difference had declined to 1 % and become statistically insignificant.

**Conclusions:**

Although female PIs remain underrepresented in clinical trial leadership, the gap is narrowing, particularly in terms of early-career progression. Future research should explore the underlying causes of these trends, including bias, career–family trade-offs, and the structural and behavioral mechanisms that shape sorting across medical specialties. Additionally, recent evidence suggests that female PIs may positively influence trial performance—particularly in patient recruitment diversity and safety outcomes, which highlights the broader significance of gender equity in clinical research.

## 1 Introduction

Gender disparities in clinical trial leadership persist, with women underrepresented as principal investigators (PIs). Although previous studies highlight this imbalance, most rely on small, specialty-specific datasets, limiting generalizability ([[1], [2], [3], [4], [5]]). In addition, these studies do not examine how representation may have evolved over time. Moreover, beyond static representation metrics, disparities in career progression could lead to long-term inequities in research leadership, making it essential to examine gender differences not only in trial leadership rates but also in terms of career progression. Understanding gender representation in clinical trials is particularly important given the role of PIs in shaping research priorities, ensuring patient diversity, and overseeing study execution ([[6], [7], [8], [9], [10]]).

In this study, I used data from ClinicalTrials.gov, the most comprehensive clinical trial registry worldwide, to provide a large-scale analysis of gender representation and career progression among clinical trial PIs. The analysis covers nearly 160,000 interventional trials initiated between 2010 and 2023. First, I assess trends in the representation of female PIs, their differences between specialties, types of intervention, and trial phases. Second, I examine differences in early-stage career trajectories between male and female PIs by tracking the number of trials led from each researcher's first recorded trial. Finally, I assess whether gender disparities in trial leadership have narrowed over time. By addressing both representation and career progression, this study offers new insights into the evolving landscape of gender equity in clinical trial leadership.

## 2 Methods

### 2.1 Data source and main data features

I used information from clinical trials submitted to ClinicalTrials.gov. Data were obtained from the Aggregate Analysis of ClinicalTrials.gov website, which restructures and standardizes trial data for analysis [11]. I restricted the sample to trials initiated between 2010 and 2023. The start year reflects improvements in data completeness following pro-transparency policies in the 2000s, while 2023 was the last year with largely complete records at the time of analysis.

Of the 285,922 trials available in the full data, 161,294 reported information in the field assigned to the identity of PIs. There was enough information to infer the gender of the PI for 156,539 trials. Of these, 91.5 % were associated with a single PI; 8.5 % with more than one.

The data set covers trials that test various types of interventions, such as drugs, biologicals, devices, and behavioral treatments. There is also information about the therapeutic areas associated with each trial (in the form of MeSH codes), the trial phase (when applicable), and the lead sponsors. Detailed descriptive statistics of all variables entering the analyses are presented in Table S.1.

### 2.2 PI gender codification

To infer the gender of each PI, I used genderapi.io, an algorithmic tool that infers gender from first names using a vast database that correlates names with their predominant gender associations across diverse cultures and regions. This algorithm returns a top gender prediction (male or female) along with a probability estimate. I selected this tool on the basis of published evidence highlighting it as best-in-class, reporting accuracy rates above 97 % and overall error rates below 2 % in comparative evaluations [12].

To implement this procedure, I first standardized and cleaned the text fields reporting PI names. Non-name entries and initials were removed, and resulting PI names split into first and last names. The distribution of the probability that an investigator is female is illustrated in Figure S.1. An important feature of this distribution is the significant concentration at its extremes; 82 % of the data reside outside the central interval (0.1,0.9), suggesting that gender codification is carried out largely with a high degree of confidence. Moreover, as shown in the examples of Figure S1, the algorithm appears capable of correctly classifying male and female names that are not from the Global West, further supporting its applicability across diverse cultural contexts.

For the main analysis, a PI is codified as female if the probability reported by the algorithm of being female is 0.5 or greater. This threshold assigns gender according to the most likely of two mutually exclusive outcomes, male or female. (Like the rest of the literature, I abstract from gender-fluidity considerations.) Using this specification, approximately a third of the trials (56,883 of 156,539) in the sample are led by a female PI. This estimate is closely aligned with the analog figure reported by the only other large-scale study that I was able to identify [13], which is 32.4 %.

It is worth noting that the specific choice of probability threshold has minimal practical impact on the results, provided it remains within a reasonable range. This occurs due to the strongly bimodal distribution of probabilities (Figure S1). To illustrate this, Figure S.2 plots the percentage of trials classified as female-led as the classification threshold varies. As the probability threshold increases, the curve gradually decreases without abrupt changes in the interior range of thresholds. This pattern indicates that the classification is stable for any sensible threshold choice. As a robustness check, I also replicate the main findings using stricter thresholds of 0.7 and 0.9.

### 2.3 Disambiguation of researcher identities

To track career progressions, a key step was to disambiguate the identity of each investigator. This step was necessary because the original data do not include individual-level identifiers. This limitation forced me to rely on free-text entries for the PIs’ names and institutions, which have inconsistent formats. Common issues included misspellings (e.g., “Kathrin Milbury” vs. “Kathryn Milbury”), inconsistent reporting of degrees and titles (e.g., “Kathleen Lyons, ScD” vs. “Kathleen Lyons, ScD, OTR/L″), and variations in affiliations (e.g., “Ohio State University Comprehensive Cancer Center” vs. “The Ohio State University”). Additional details for the affiliation disambiguation process are presented in Section B of the Supplementary Material.

I used a fuzzy text matching procedure to determine when slightly different text entries corresponded to the same individual. The algorithm relies on the Jaccard similarity score, which is a measure of text similarity. For individuals with different affiliations, names were considered the same if their Jaccard similarity distance was 0.975 or higher. If the reported affiliations were the same, a lower threshold of 0.95 was used.

### 2.4 Investigator cohorts

To investigate temporal trends in career progression, two concepts are introduced: “inaugural trial” and “investigator cohort.” An inaugural trial is the first trial that an investigator leads as principal investigator, marking their observable entry into trial leadership. An investigator cohort is the set of all investigators whose inaugural trial was initiated in a given year. For example, the 2013 cohort includes all investigators whose inaugural trial began in 2013. Based on these concepts, I define a third concept: “entry into the field.” This refers to the year in which an investigator's inaugural trial was initiated and serves as the reference point for measuring the vigor of early-stage career progression.

The concepts of inaugural trial and investigator cohort have both practical and conceptual limitations, which I discuss more fully in the Limitations section. Inaugural trials are identified from the full AACT dataset, including trials initiated before my 2010–2023 study period, with many going back one or two decades. The main practical limitation is that, before my sample period, transparency issues resulted in very low registration rates, and it was not until regulatory changes between 2005 and 2009 that this matter improved substantially ([[14], [15], [16]]). As a result, for some investigators —particularly those who began their careers before the mid-2000s— I may systematically overestimate the year of their inaugural trial. Conceptually, there are valid concerns about whether entry into the field should be measured through inaugural trials, given that researchers may enter clinical research in other capacities, such as co-investigators, collaborators, or consultants, before appearing as principal investigators.

I adopt these concepts while acknowledging their limitations. My approach is motivated by empirical pragmatism: these definitions can be implemented at scale, which is a central objective of this study. In adopting this approach, I find some reassurance in the observation that there is no obvious reason why either the practical or conceptual limitations would affect male and female investigators differently. Under the assumption that these limitations introduce similarly distributed measurement errors for investigators of each gender, they would not bias my main results, which focus on differential trends between male and female investigators over time.

### 2.5 Outcomes

I examine two primary outcomes. The first is an indicator for whether a clinical trial is led by a female PI. This measure equals one if at least half of the codified PIs associated with the trial are female, and zero otherwise. This type of outcome is standard in the literature and has been widely used to assess female representation in clinical trial leadership ([[1], [2], [3], [4], [5],^13^]).

The second outcome aims to capture gender differences in early-stage career progression. It corresponds to the cumulative number of clinical trials initiated by a PI within a fixed time horizon after entering the field (including the inaugural trial). I consider three time horizons: three, five, and seven years. These quantities track the intensity and continuity of participation in trial leadership and, therefore, function as a proxy for early career productivity. For example, if one researcher initiates three trials within five years of entering the field while another initiates only one, this would be interpreted as the first researcher having a more productive early career stage. Similarly, if within the same time horizon one group of researchers averages 1.6 trials while another group averages 1.5 trials, that would be interpreted as the first group enjoying, on average, greater early career momentum.

An important caveat is that both outcomes may not fully capture all dimensions of quality, scale, or impact. This is because both measures treat all trials as equivalent units. In other words, neither the representation nor the career progression outcomes distinguish between small pilot studies and pivotal Phase III programs, between incremental and novel clinical questions, or between trials that succeed and those that fail. This measurement problem would imply, for example, that if female PIs on average lead trials with greater scientific impact, then the roughly one-third female PI share documented in the literature may underestimate the true influence of women in clinical research.

This limitation, however, is neither unique to this study nor to clinical trials. Where research output is heterogeneous and difficult to quantify, there is a tendency to converge on count-based metrics as pragmatic indicators of productivity. For example, patent counts are used to measure inventor productivity, while publication counts are an important input for academic promotion decisions in many disciplines ([17,18]). Although imperfect, these measures provide transparent and reproducible foundations for comparative analysis.

Importantly, the limitations of count-based measures are less problematic for this study than for cross-sectional analyses. Rather than assessing absolute levels of representation or productivity, the primary objective here is to evaluate how gender gaps have evolved over time. Dimensions of quality, scale, or impact that are not captured by trial counts would therefore not bias the estimated evolution of these gaps, provided they affect male and female researchers similarly.

### 2.6 Statistical analyses

I perform two sets of analyses. The first is a representation analysis. This analysis assesses the likelihood that a clinical trial is led by a female PI. The second is a career trajectory analysis. This analysis characterizes gender differences in the number of trials led within fixed time horizons after entering the field.

In the representation analysis, I estimate a logistic regression model. This model relates the probability of a trial being led by a female PI to the year the trial was initiated. I control for a broad set of observable trial characteristics. The model parameters are estimated by maximum likelihood. Standard errors are clustered within therapeutic areas to account for possibly correlated errors.

The set of controls includes indicators for therapeutic area, type of intervention, sponsor category, and whether the trial lists multiple PIs. Controlling for these characteristics helps ensure that comparisons of gender representation over time are not confounded by systematic differences in the composition of trials. For example, if behavioral trials became more common over time, the raw trend would overstate progress in gender equity because these trials tend to have higher female PI representation. Including these controls helps isolate changes that more plausibly reflect evolving gender dynamics rather than changes in the types of trials being conducted.

Therapeutic areas are defined using three-digit MeSH codes. The types of intervention correspond to the categories reported in the AACT data. These include drugs, biologicals, devices, procedures, behavioral interventions, dietary supplements, diagnostic tests, radiation, and other or unreported types. The lead sponsors refer to the primary organizations responsible for the conduct of each trial. They are classified into industry, government (non-NIH), NIH, network, and other or unreported sponsors. These classifications follow the conventions of the AACT data. This set of variables denotes the most comprehensive set of controls that I could compile from the data without substantially reducing sample size.

To assess whether changes in female PI leadership were concentrated in specific types of trials or generalized across the clinical research landscape, I also perform a heterogeneity analysis. This analysis applies the same logistic regression framework described above to separate subsets of trials. These subsets are defined by therapeutic area, type of intervention, phase, and sponsor category. To assess temporal trends, I compare the results obtained from estimating the models separately in data from two subperiods, 2010–2016 versus 2017–2023.‘

The second analysis examines gender differences in early-stage career progression using the cumulative trial counts described above. I model these counts using Poisson regression specifications that relate the number of trials led within each time horizon (three, five, or seven years) to the investigator's gender and cohort of entry. From these results, I compute the female-to-male incidence rate ratio (IRR) for each cohort of investigators. This quantity denotes the relative rate at which female investigators of each cohort lead clinical trials compared to their male counterparts.

## 3 Results

### 3.1 Representation

**Fig. 1:**
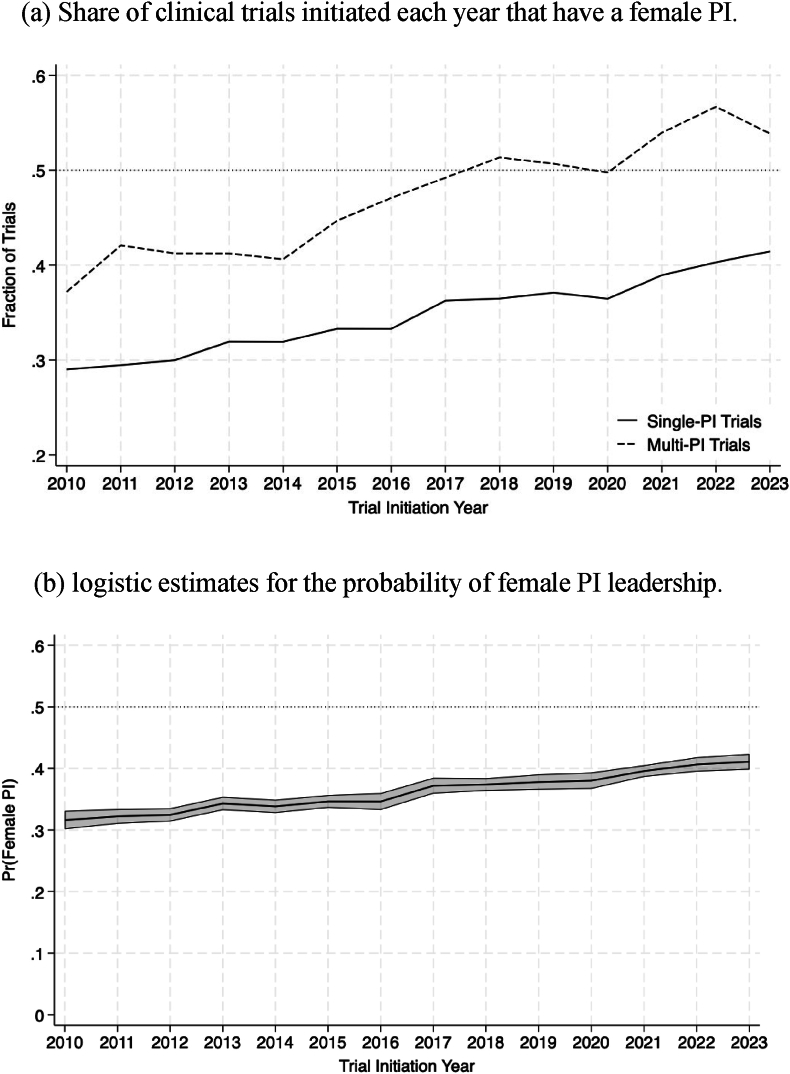
**Overall Female Representation.** Panel (a) shows the share of clinical trials initiated each year that have a female principal investigator (PI). The solid line represents single-PI trials, while the dashed line represents multiple-PI trials. Panel (b) presents the estimated probabilities that a trial initiated each year is led by a female PI. Estimates are derived from a logistic model that includes year fixed effects to capture year-specific probabilities of female PI leadership. The model also controls for single vs. multiple PIs, trial phase, sponsor type, intervention type, and therapeutic area, all included as fixed effects. 95 % confidence intervals are computed using standard errors clustered within unique therapeutic area combinations.

Fig. 1a illustrates the proportion of clinical trials initiated each year that have a female PI. The solid line represents the trend for single-PI trials, while the dashed line represents multi-PI trials. Both trends indicate a steady increase in female PI participation during the study period. For single PI trials, the share of female PIs increases from approximately 30 % in 2010 to more than 40 % by 2023. Multi-PI trials consistently exhibit a higher share of female PIs but follow a similar trajectory, increasing from around 35 % in 2010 to approximately 53 % in 2023.

Fig. 1b presents the logistic regression estimates for the probability that a trial is led by a female PI as a function of the trial's initiation year. These estimates confirm the trends of Fig. 1a. The probability that a trial is led by a female PI increases monotonically throughout the sample period, from 0.32 among trials initiated in 2010 (95 % confidence interval [CI], 0.30 to 0.33) to 0.41 among trials initiated in 2023 (95 % CI, 0.40 to 0.42).

**Fig. 2:**
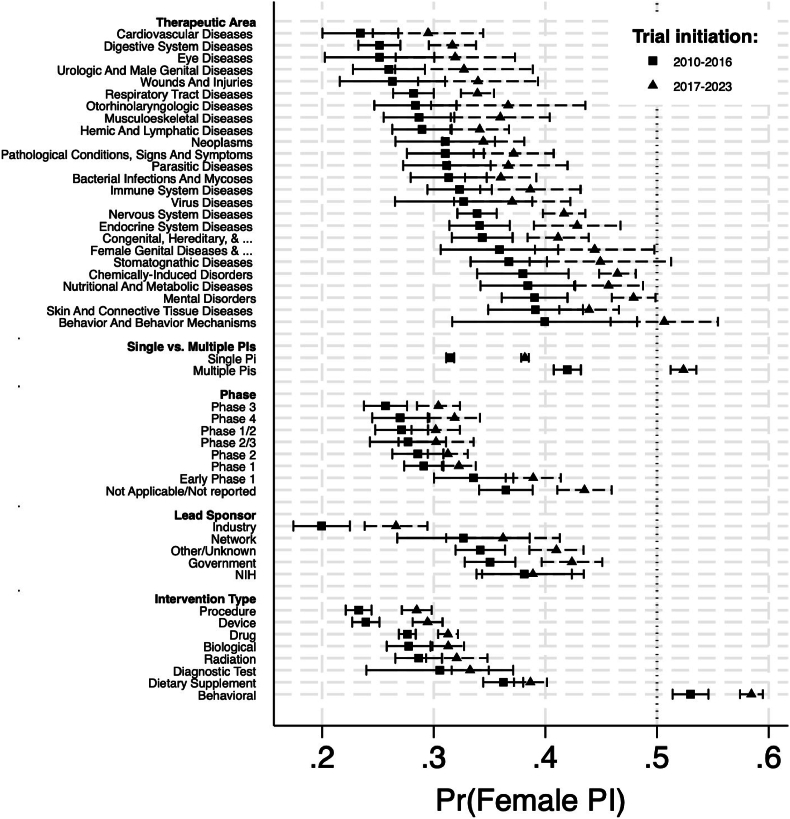
**Comparison of Female PI Leadership Across Subsamples, 2010**–**2016 versus 2017**–**2023.** To evaluate temporal changes in Female PI participation across different types of trials, I separately estimated the probability of Female PI leadership among the subsets of trials listed on the vertical axis. For each subset, I compared trials initiated during two periods, 2010–2016 versus 2017–2023. Markers denote estimated probabilities of Female PI leadership and bars denote their respective 95 % CIs. The therapeutic area categories correspond to three-digit MeSH headings, which serve as standardized therapeutic area groupings. Two headings are truncated in the figure: Congenital, Hereditary, and Neonatal Diseases and Abnormalities and Female Genital Diseases and Pregnancy Complications.

To assess whether the observed increase in female PI leadership occurred uniformly or was concentrated in specific areas of clinical research, I next examined patterns across key trial characteristics. Fig. 2 presents estimated probabilities of female PI leadership for distinct subsets of trials defined by therapeutic area, intervention type, phase, and sponsor category. Each panel compares trials initiated during 2010–2016 (orange markers) and 2017–2023 (green markers). Overall, the results show that the rise in female participation was broadly generalized rather than confined to particular trial types, with gains observed across nearly all categories.

When evaluated as proportional changes, the largest increases in female PI participation were observed in several key categories. Among intervention types, medical device trials exhibited the most substantial increase, with a 23.1 % increase (from 0.239 to 0.470, p < 0.05), followed closely by medical procedures trials, with a 22.3 % increase (from 0.233 to 0.456, p < 0.05). Within therapeutic areas, Eye Diseases trials experienced a notable 19.8 % increase (from 0.252 to 0.450), although this change was not statistically significant. Musculoskeletal disease trials also saw a significant increase with a 19.8 % increase (from 0.287 to 0.483), although not statistically significant. Other categories with considerable increases include Phase 3 trials with an increase of 18.4 % (from 0.257 to 0.441, *p <* 0.05), Phase 4 trials with an increase of 18.1 % (from 0.270 to 0.451, p < 0.05). Despite these increases, female PIs remain underrepresented in all but one type of trials, i.e., behavioral intervention trials. The analysis also identifies a decrease in female PI participation among some lead sponsors. Network-sponsored trials exhibited a proportional decrease of 7.5 % (from 0.327 to 0.252), and NIH-sponsored trials saw a decrease of 3.6 % (from 0.381 to 0.345); however, these decreases were not statistically significant.

As a complement to these representation patterns, I also analyzed the composition of trial leadership teams by size (i.e., number of PIs) and gender. The results, summarized in Table S.2, indicate that team structure has remained broadly stable over time when comparing trials initiated in the first half of the sample (2010–2016) to those in the second half (2017–2023). The proportion of single-PI trials remains nearly unchanged at roughly 91–92 percent across both periods, and the distributions of teams with two, three, or four or more PIs also exhibit minimal variation. Similarly, the proportions of all-male and all-female PI teams of different sizes do not change substantially. Instead, what drives the overall increase in female PI participation is a uniform rise across single-PI and gender-mixed teams across all sizes. Consistent with the patterns in Fig. 1a, these results suggest that the growth in female representation reflects broad-based changes across the clinical trial landscape rather than compositional shifts in team structure.

### 3.2 Career trajectories

The positive trend in female representation among clinical trial PIs documented above could be explained in two ways. On the one hand, the trend could stem from an improvement of female researchers’ career trajectories relative to those of male counterparts. That is, an increase in the probability that female researchers will lead additional trials. On the other hand, the positive trend in representation could be explained by an increase in the number of female researchers entering the field, coupled with unchanged or even worsened prospects for the development of their trialist careers.

**Fig. 3:**
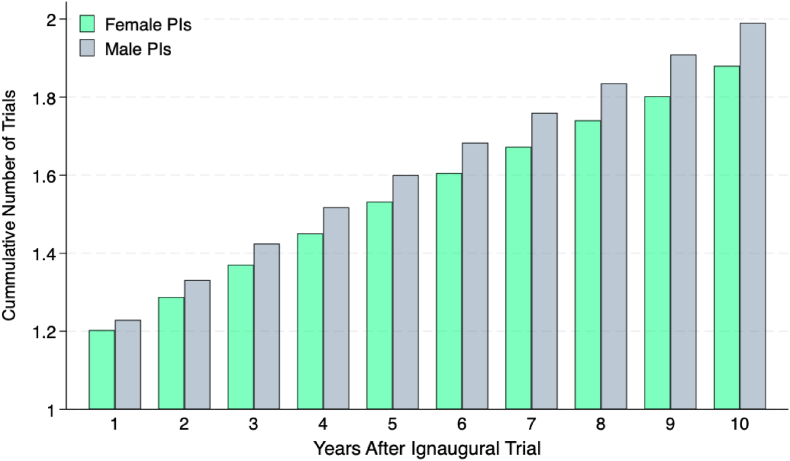
**Cumulative Number of Trials Led by Female and Male Principal PIs Over Time.** This figure illustrates the cumulative number of clinical trials led by female (green bars) and male (gray bars) PIs, as a function of the number of years following each investigator's first recorded (inaugural) trial within the data source. The horizontal axis describes the number of years after the inaugural trial; the vertical axis, the cumulative number of trials (averaged across all investigators of each gender).

To investigate this issue, I analyze the career trajectories of female PIs relative to their male counterparts. I characterize each researcher's career trajectories through the number of trials led he or she leads as PI within a fixed time horizon, as described formally in a section above. Fig. 3 describes these trial counts, averaged for male and female PIs whose inaugural trial occurred within the study period. The horizontal axis shows different time horizons, i.e., the number of years elapsed since an investigator's inaugural trial.

On average, within the first year following their inaugural trial, PIs lead 1.219 trials (95 % CI: 1.214–1.223). Since this count includes the inaugural trial itself, this average is consistent with approximately 22 % of PIs initiating a second trial within the first year, though other distributions of trial initiation across investigators could also produce this mean. This number increases to 1.954 (95 % CI: 1.927–1.980) a decade after their inaugural trial. The increment of 0.954 indicates that the average PI initiates roughly one additional trial within ten years of entering the field.

A persistent gender gap emerges across all time horizons: male investigators consistently lead more trials than their female counterparts. Although the average male PI progresses from 1.228 cumulative trials (95 % CI: 1.222–1.234) at the one-year mark to 1.990 (95 % CI: 1.956–2.025) at the ten-year mark, the average female PI follows a trajectory from 1.203 trials (95 % CI: 1.196–1.210) to 1.879 (95 % CI: 1.837–1.922) over the same period.

**Fig. 4:**
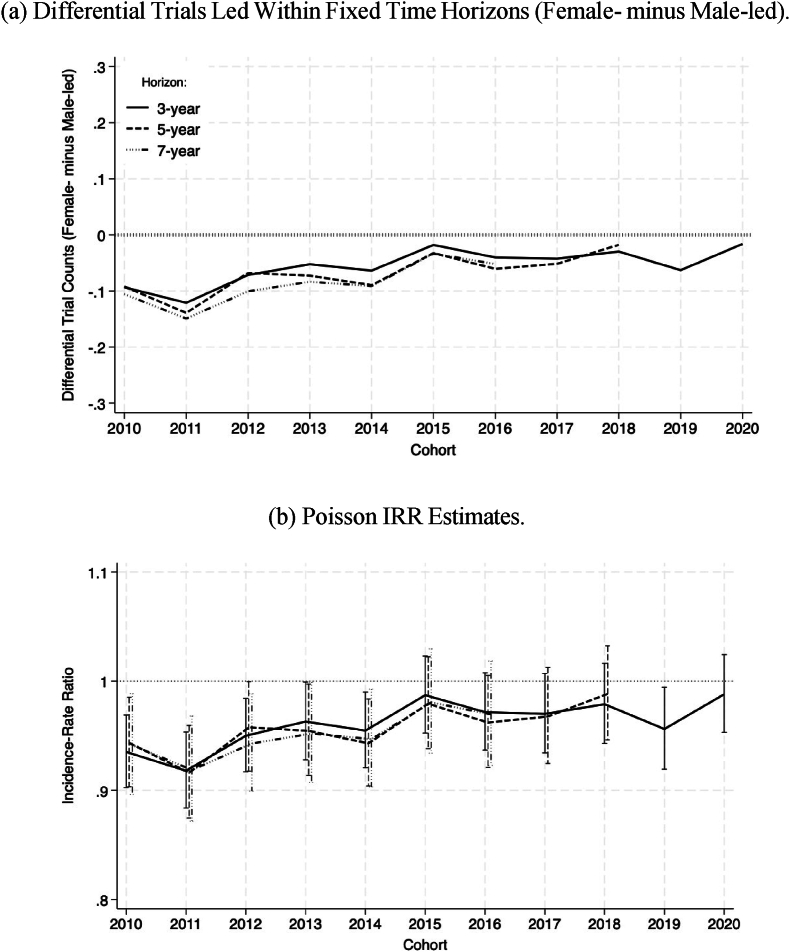
**Gender Differences in Early-Stage Career Trajectories.** Panel (a) shows the differential number of trials led by PIs within each time horizon, calculated as the average number of trials led by female PIs of each cohort minus that of trials led by male PIs. A negative differential indicates that, on average, male PIs lead more trials than female PIs. Panel (b) presents Poisson regression estimates of the incidence rate ratio (IRR) for the cumulative number of trials led by male and female PIs within each cohort. An IRR of 1 indicates that there is no systematic gender difference, while an IRR below 1 suggests that female PIs lead fewer trials on average than male PIs. Conversely, an IRR above 1 would indicate that female PIs systematically lead more trials than their male counterparts.

To assess how these trends have evolved over time, I analyze the total number of trials led within fixed time horizons for researchers of each cohort. The solid line in Fig. 4a plots the difference in the average number of trials led by female PIs minus that of trials led by male PIs within a 3-year horizon. For the 2010 cohort, this difference is approximately −0.1. This number indicates that, on average, male PIs led about 0.1 more trials than their female counterparts (approximately 7 % of the baseline). This differential remains negative for all cohorts until 2020. This pattern means that, on average, male PIs consistently lead more trials than female PIs in the early years of their careers. Importantly, the gap has progressively narrowed over time, with the differential decreasing to approximately −0.015 for the 2020 cohort (about 1 % of the baseline).

The dashed and semi-dashed lines in Fig. 4a extend the analysis by examining cumulative trials within 5- and 7-year horizons, respectively. Notably, these trends closely follow the 3-year horizon trend. This aspect of the data suggests that, while present, differences in career trajectories between male and female PIs emerge early in their careers and tend to persist over time.

To assess the statistical significance of these patterns, I estimate Poisson regression models using as an outcome the total number of trials led by each PI within a fixed time horizon. Results are presented in Fig. 4b in the form of cohort-specific incidence rate ratios (IRR). An IRR of 1 indicates that there is no systematic difference between the cumulative number of trials led by male and female researchers of each cohort, while an IRR below 1 suggests that female PIs lead systematically fewer trials than their male counterparts.

The temporal trends outlined by the estimated IRRs closely mirror those derived from the raw averages presented in Fig. 4a, across the three time horizons. The key insight from these formal results is that gender differences in the progression of early-stage careers have steadily decreased since 2010, stabilizing around 2015. At this point, estimates suggest that male PIs lead approximately 5 % more trials than female PIs. However, from 2015 onward, these differences lack statistical significance and I cannot reject the hypothesis of no gender differences.

## 4 Discussion

This analysis provides two key insights into gender disparities among clinical trial principal investigators (PIs). First, I confirm previous findings that about a third of clinical trials have a female PI [13]. Like most previous work in the same space ([[1], [2], [3], [4], [5],^13^]), I interpret this result as consistent with the under-representation of women in clinical trial leadership.

This interpretation is uncontroversial, yet it involves an element of subjectivity. The literature lacks clarity on the appropriate benchmark against which under-representation should be measured. True gender equity, under- or over-representation ought to be assessed relative to the gender composition of the pool of qualified and available researchers to lead clinical trials. But that pool is empirically difficult to grasp. A leading element of this difficulty is the so-called “leaky pipeline” [19]: while women outnumber men in many medical and biomedical training programs, their representation decreases in higher academic ranks and leadership positions. Consequently, even a 50/50 distribution of male and female PIs in trial leadership may not reflect complete gender equity, as it could still mask underlying imbalances in advancement opportunities throughout the career pipeline.

Unlike most previous studies that rely on smaller samples focused on specific therapeutic areas, the analysis covers the full set of clinical trials submitted to ClinicalTrials.gov.^1^ This includes nearly 160,000 trials initiated between 2010 and 2023. Within this comprehensive dataset, consistent with previous findings, I find that the representation of female PIs is lowest in cardiovascular trials, where only about one in five trials has a female PI. It is highest in trials targeting behavioral-related conditions, where one in two trials has a female PI. I document the encouraging finding that female representation has increased in most therapeutic areas and trial types over the 14 years covered by the sample.

Although representation statistics are the obvious starting point for examining gender disparities in clinical trial leadership, they do not fully capture the long-term sustainability of gender equity. To illustrate this point, consider a hypothetical scenario in which female PIs lead half of all trials. By standard representation metrics –and assuming equally sized pools of qualified and available male and female researchers– this would indicate full gender parity. However, this parity would be superficial if female PIs led only a single trial before exiting the field, while male PIs continued to establish long-term careers involving multiple trials. In such an extreme scenario, female researchers would be effectively confined to early-career leadership roles. This would limit their opportunities to achieve senior positions, shape clinical research priorities, and mentor future generations of scientists. Thus, achieving gender equity in trial leadership requires not only equal representation, but also the capacity for sustained career advancement.

My second set of findings directly addresses this issue by examining the career trajectories of male and female PIs. Over a 10-year horizon following an investigator's first recorded trial, male PIs consistently lead more trials than their female counterparts. However, my evidence suggests that this gender gap has progressively narrowed over time. Among researchers who entered the field in 2010, male PIs led approximately 7 % more trials than female PIs within their first three years. By 2020, this difference had decreased to just 1 %. Regression estimates further suggest that this difference stabilized around 2015. Confidence intervals have widened to the extent that I can no longer reject the hypothesis of no systematic gender-based disparities in early career trial leadership. These results indicate that while female PIs may still encounter structural barriers in accessing leadership roles in clinical trials, the trajectory is improving. This reflects a positive trend toward greater gender equity in career progression.

Observers have suggested that the under-representation of women in clinical trial leadership roles may be driven, at least in part, by biases that contribute to discrimination [6]. The presence of such biases is difficult to dispute, given extensive anecdotal evidence—for example, the prevalence of “manels” (i.e., all-male panels) at leading medical conferences [20]. However, it is important to recognize that other factors can also play a role in shaping the gender disparity. One key factor is the challenge of balancing career and family, which imposes greater professional costs on women than on men [21]. Another factor relates to differences in the gender composition and career progression of physicians across medical specialties. For example, in the United States (2022–2023), women outnumber men by almost a three-to-one ratio among pediatric residents, while the ratio is reversed among cardiology residents [22]. These patterns are shaped by the interaction of multiple forces. These include individual interests, mentorship and role-model exposure, professional networks, social mechanisms such as conformity and homophily, among others. Together, these dynamics may create enduring asymmetries in how men and women sort into specialties and career trajectories. It is important to keep in mind that these mechanics would remain present even if the above-mentioned biases and labor market frictions disappeared.

A critical avenue for future research is to disentangle the relative contributions of bias, career–family trade-offs, and the structural and behavioral mechanisms that shape sorting across medical specialties. A more precise understanding of how individual interests interact with mentorship, institutional support, and professional networks could help inform policies that promote greater gender equity in clinical trial leadership. Furthermore, recent evidence links female PIs with more diverse patient recruitment [8] and a lower prevalence of severe adverse events in clinical trials [23]. These findings suggest that, beyond being a matter of equity, female representation in clinical trial leadership may also matter for clinical trial performance.

## Limitations

Several limitations of this study should be acknowledged. Some were discussed earlier in the text.

First, gender was inferred algorithmically from first names. The identities of the investigators were disambiguated using fuzzy matching techniques. These procedures, described in the *Methods* section, introduce the possibility of misclassification or name conflation. This is particularly true for investigators with culturally ambiguous names or inconsistently reported affiliations. The accuracy rates for the gender inference tool are high. Identity errors likely add random noise rather than systematic bias. However, these remain measurement imperfections inherent to large-scale registry data. Furthermore, the algorithmic approach used in this study classifies investigators into binary gender categories (male or female) and does not capture the full spectrum of gender identity. This limitation reflects both the constraints of name-based inference methods and the absence of self-reported gender identity data in clinical trial registries.

Second, both outcome measures –female representation and early-career progression– treat all clinical trials as equivalent units. They do not account for variation in scale, novelty, or scientific impact. As discussed in the *Outcomes* section, this constraint reflects a broader challenge in measuring productivity in research settings. Count-based indicators are often the only scalable and reproducible option. However, my analysis focuses on changes in gender gaps rather than absolute levels. Therefore, these limitations are unlikely to bias the main findings, provided that these unobserved trial characteristics affect male and female investigators similarly.

Third, the concept of “entry into the field” provides an approximation of the onset of each researcher's career. I define it as the investigator's first recorded trial as PI. Some researchers may have participated in earlier trials in non-leadership roles that are unobserved in the data. As noted previously, this abstraction may blur differences in underlying experience levels. I have nevertheless adopted it because it enables consistent measurement across time and cohorts.

Finally, the registry data do not capture contextual factors such as mentorship, institutional support, or funding environments. These factors likely shape both representation and career progression. These unobserved mechanisms remain important topics for future research on gender equity in clinical trial leadership.

## Declaration of competing interest

The authors declare that they have no known competing financial interests or personal relationships that could have appeared to influence the work reported in this paper.

## Data Availability

Data will be made available on request.

## Footnotes

1 A notable exception is [13], who also use a large sample of trials from ClinicalTrials.gov.
