# Supplementary material for: Representation and career trajectories of female principal investigators in clinical trials, 2010–2023
Source: Contemp Clin Trials Commun. 2025 Oct 30;48:101566. doi: 10.1016/j.conctc.2025.101566 (PMC12634453; doi:10.1016/j.conctc.2025.101566)
Supplement: Multimedia component 1 [file mmc1.docx]

**SUPPLEMENTAL MATERIALS**

# A Additional Displays

**Table S.1. Descriptive Statistics of Clinical Trials Dataset.** This table provides descriptive statistics of the clinical trials dataset, including information on the principal investigator (PI) structure and gender, therapeutic areas, types of interventions, phases of trials, and lead sponsors. The trial sample was downloaded from the Aggregate Analysis of ClinicalTrials.gov database in April 2023 and contains information on 156,539 interventional trials initiated between 2010 and 2023. In some categories, percentages do not sum up to 100% due to the possibility of overlap among categories.

| (a) Principal Investigator (PI) Structure and Gender | | Count | Proportion (%) |
| --- | --- | --- | --- |
| Single PI Trials (Female PI) | | 50,432 | 32.2% |
| Single PI Trials (Male PI) | | 92,527 | 59.1% |
| Multi PI Trials (50% or more Female PIs) | | 6,451 | 4.1% |
| Multi PI Trials (less than 50% Female PIs)  (b) Therapeutic Areas | | 7,129 | 4.6% |
| Bacterial Infections and Mycoses (C01) | | 10,147 | 6.5% |
| Virus Diseases (C02) | | 5,905 | 3.8% |
| Parasitic Diseases (C03) | | 725 | 0.5% |
| Neoplasms (C04) | | 29,614 | 18.9% |
| Musculoskeletal Diseases (C05) | | 7,739 | 4.9% |
| Digestive System Diseases (C06) | | 11,862 | 7.6% |
| Stomatognathic Diseases (C07) | | 3,531 | 2.3% |
| Respiratory Tract Diseases (C08) | | 12,727 | 8.1% |
| Otorhinolaryngologic Diseases (C09) | | 2,238 | 1.4% |
| Nervous System Diseases (C10) | | 25,191 | 16.1% |
| Eye Diseases (C11) | | 3,460 | 2.2% |
| Urologic and Male Genital Diseases (C12) | | 7,979 | 5.1% |
| Female Genital Diseases and Pregnancy Complications (C13) | | 9,460 | 6.0% |
| Cardiovascular Diseases (C14) | | 17,101 | 10.9% |
| Hemic and Lymphatic Diseases (C15) | | 7,545 | 4.8% |
| Congenital, Hereditary, and Neonatal Diseases and Abnormalities (C16) | | 5,521 | 3.5% |
| Endocrine System Diseases (C17) | | 9,204 | 5.9% |
| Nutritional and Metabolic Diseases (C18) | | 12,852 | 8.2% |
| Immune System Diseases (C19) | | 8,158 | 5.2% |
| Skin and Connective Tissue Diseases (C20) | | 10,784 | 6.9% |
| Pathological Conditions, Signs and Symptoms (C23) | | 40,371 | 25.8% |
| Stomatognathic Diseases (C25) | | 3,210 | 2.0% |
| Respiratory Tract Diseases (C26) | | 6,173 | 3.9% |
| Behavior and Behavior Mechanisms (F01) | | 5,347 | 3.4% |
| Mental Disorders (F03) | | 14,650 | 9.4% |
| Other/Unreported  (c) Types of Interventions | | 28,417 | 18.2% |
| Behavioral | | 25,993 | 16.6% |
| Biological | | 8,557 | 5.5% |
| Device | | 25,402 | 16.2% |
| Diagnostic Test | | 2,809 | 1.8% |
| Dietary Supplement | | 7,296 | 4.7% |
| Drug | | 56,224 | 35.9% |
| Procedure | | 16,553 | 10.6% |
| Radiation | | 3,881 | 2.5% |
| Other/Unreported  (d) Phases of Trials | | 9,824 | 6.3% |
| Early Phase 1 | | 2,732 | 1.8% |
| Phase 1 | | 12,371 | 7.9% |
| Phase 1/Phase 2 | | 5,512 | 3.5% |
| Phase 2 | | 19,465 | 12.4% |
| Phase 2/Phase 3 | | 2,453 | 1.6% |
| Phase 3 | | 8,391 | 5.4% |
| Phase 4 | | 12,401 | 7.9% |
| Unreported/Not Applicable  (e) Lead Sponsors | | 93,214 | 59.5% |
| Industry | 18,871 | | 12.1% |
| Government | 5,764 | | 3.7% |
| NIH | 1,985 | | 1.3% |
| Network | 914 | | 0.6% |
| Other/Unreported | 129,005 | | 82.4% |

**Figure S.1. Probability Distribution of Principal Investigator First Names Corresponding to Females.** This figure shows the probability that the first name of a clinical trial principal investigator corresponds to a female person, along with the density given by the number of trials in the full sample associated with each probability. The main point of the figure is that the bulk of the mass is either close to zero or close to 1, meaning that the bulk of gender classification is carried out with little classification uncertainty. In fact, 82% of the data are outside the central interval ranging from 0.1 to 0.9. Another important point illustrated by examples of names is that the algorithm seems to accurately capture names that are not from the Western world (e.g., Jeffrey, Gunnar, Matthias, Vivek) and classify them correctly as either male or female. Additionally, names that are gender ambiguous, such as Andrea, are assigned a probability of being female that is close to 0.5, reflecting the uncertainty in gender classification for these names.


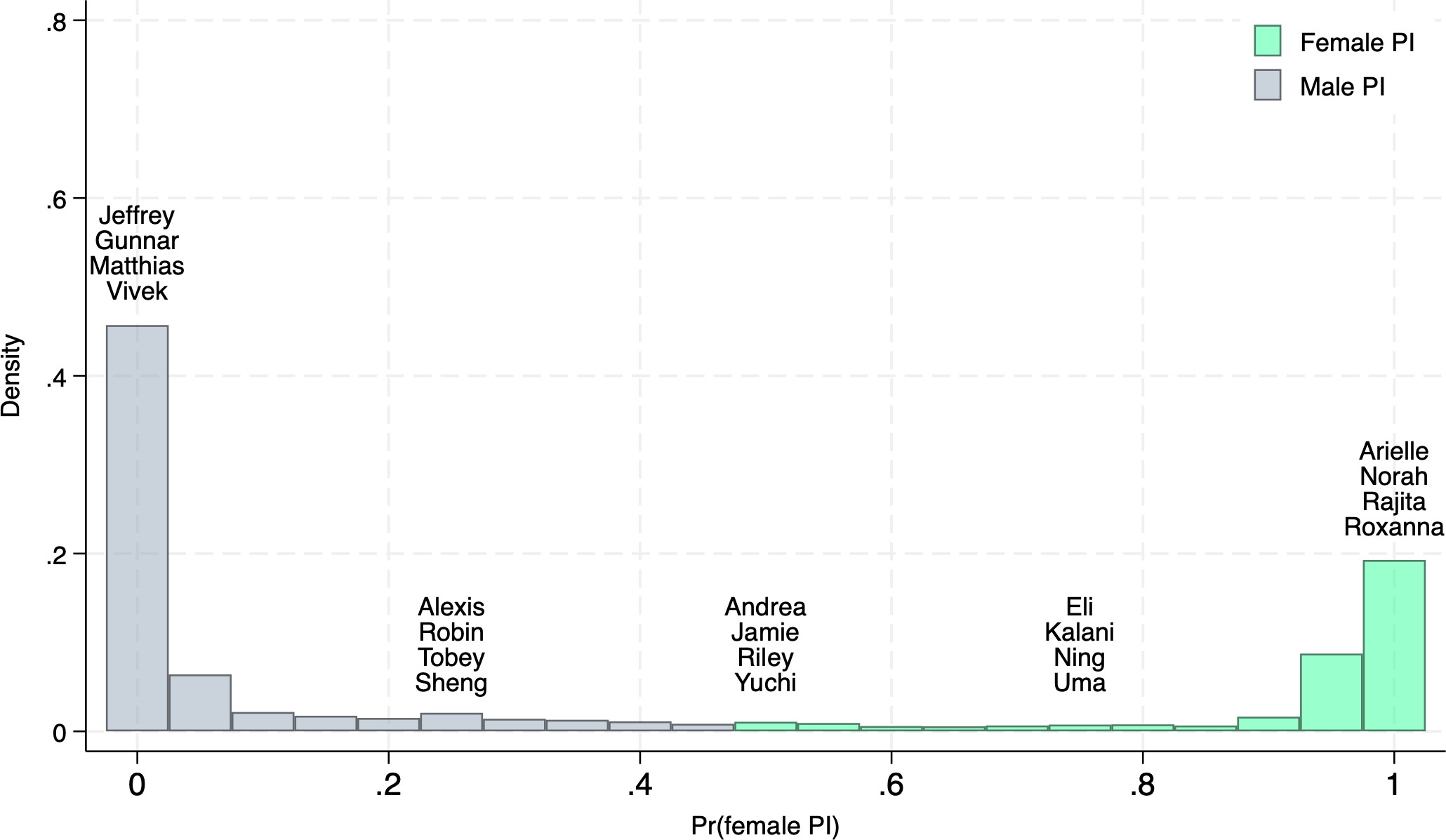


**Figure S.2. Sensitivity of Female-PI Classification to Probability Thresholds.** This figure plots the fraction of trials classified as having a female principal investigator (PI) against the probability threshold used to determine classification. In the main analysis, I use a threshold of 0.5, which naturally assigns gender based on the higher of the two predicted probabilities (female vs. male). To test robustness, I also report results using thresholds of 0.7 and 0.9 (vertical dashed lines). The curve is relatively flat across the interior range of thresholds, reflecting the fact that the underlying probability distribution of names (Figure [Figure S.1](#_bookmark30)) places most mass near 0 or 1, with relatively few names assigned probabilities in the intermediate range. This implies that the classification of trials as female-led is not highly sensitive to the specific choice of threshold..


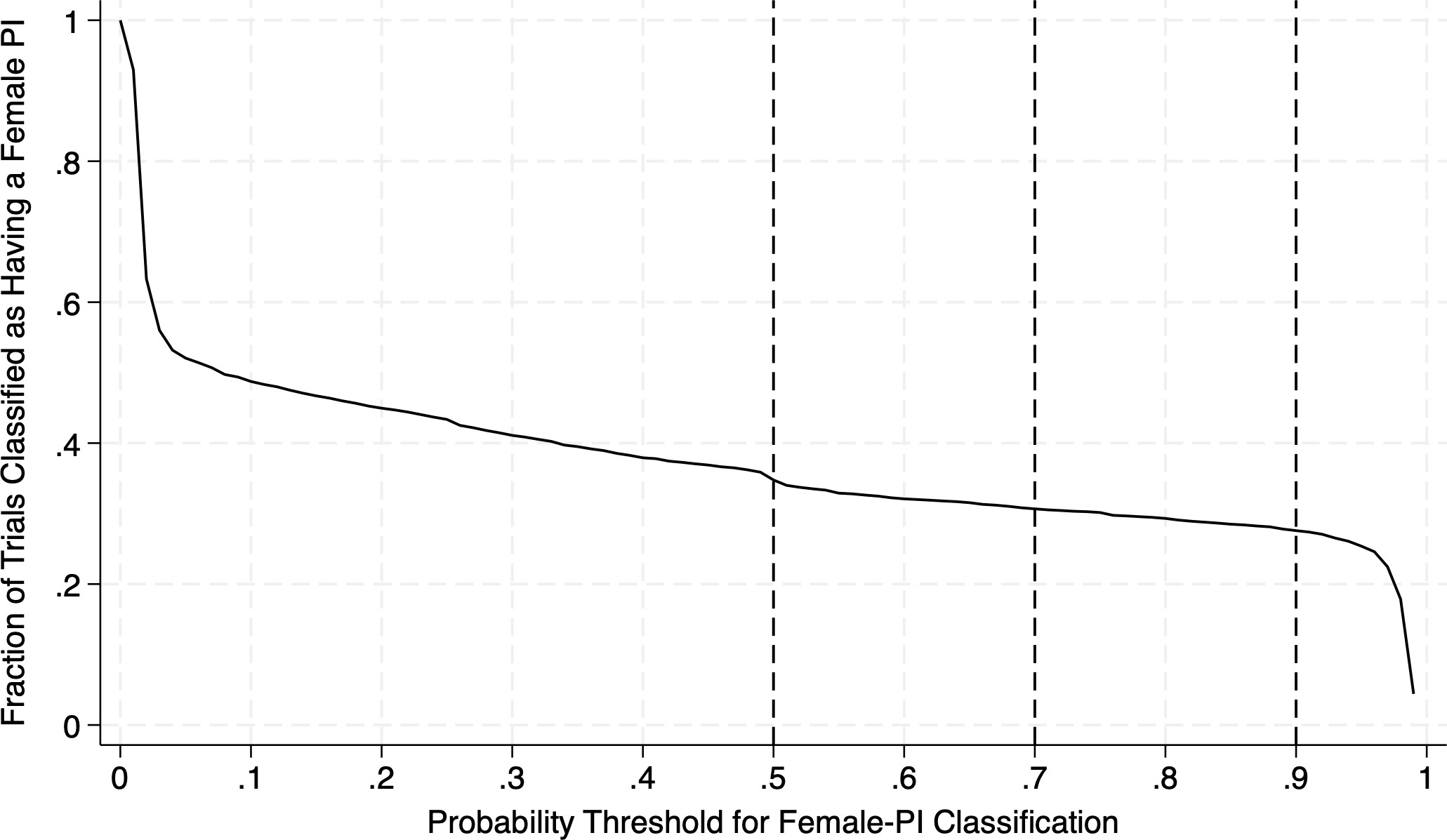


**Table S.2. Distribution of Clinical Trials by Number of Principal Investigators and Gender Composition.** In this table, each cell reports the proportion of trials falling into the specified category, based on the number of listed principal investigators (PIs) and their gender composition. Trials are grouped by initiation period (2010–2016 and 2017–2023). “Share of trials” indicates the proportion of all trials in each PI-count category. “Share of female PIs” represents the proportion of female investigators among all PIs in those trials. “Share of all-male PI teams” and “Share of all-female PI teams” indicate the fraction of trials composed exclusively of male or female investigators, respectively. For single-PI trials, by construction, these two measures coincide with the gender of the sole PI.

Number of Principal Investigators (PIs)

|  |  | 1 | 2 | 3 | 4+ | Total |  |
| --- | --- | --- | --- | --- | --- | --- | --- |
|  | (a) Trials initiated 2010–2016 | | | |  |  | |
| Share of trials | 0.91 0.06 0.01 | | | | 0.02 | 0.25 | |
| Share of female PIs | 0.31 0.33 0.33 | | | | 0.26 | 0.31 | |
| Share of all-male PI teams 0.69 0.24 0.12 | | | | | 0.03 | 0.27 | |
| Share of all-female PI teams 0.31 0.07 0.03 | | | | | 0.00 | 0.10 | |
| (b) Trials initiated 2017–2023 | | | | |  |  | |
| Share of trials 0.92 0.06 0.01 | | | | | 0.01 | 0.25 | |
| Share of female PIs 0.38 0.39 0.38 | | | | | 0.35 | 0.38 | |
| Share of all-male PI teams 0.62 0.20 0.11 | | | | | 0.03 | 0.24 | |
| Share of all-female PI teams 0.38 0.10 0.04 | | | | | 0.00 | 0.13 | |

1. Overall, 2010–2023

| Share of trials | 0.92 0.06 0.01 | 0.01 | 0.25 |
| --- | --- | --- | --- |
| Share of female PIs | 0.35 0.36 0.35 | 0.31 | 0.34 |
| Share of all-male PI teams | 0.65 0.22 0.12 | 0.03 | 0.25 |
| Share of all-female PI teams | 0.35 0.08 0.03 | 0.00 | 0.12 |

**Figure S.3. Robustness of Female PI Representation Estimates to Alternative Classification Thresholds.** This figure reproduces the logistic regression estimates shown in [Figure 1](#_bookmark25)b, but classifies principal investigators (PIs) as female using higher probability thresholds of 0.7 (Panel a) and 0.9 (Panel b). All other estimation procedures are as in the main analysis (threshold = 0.5).

1. Logistic estimates with Threshold = 0.7.


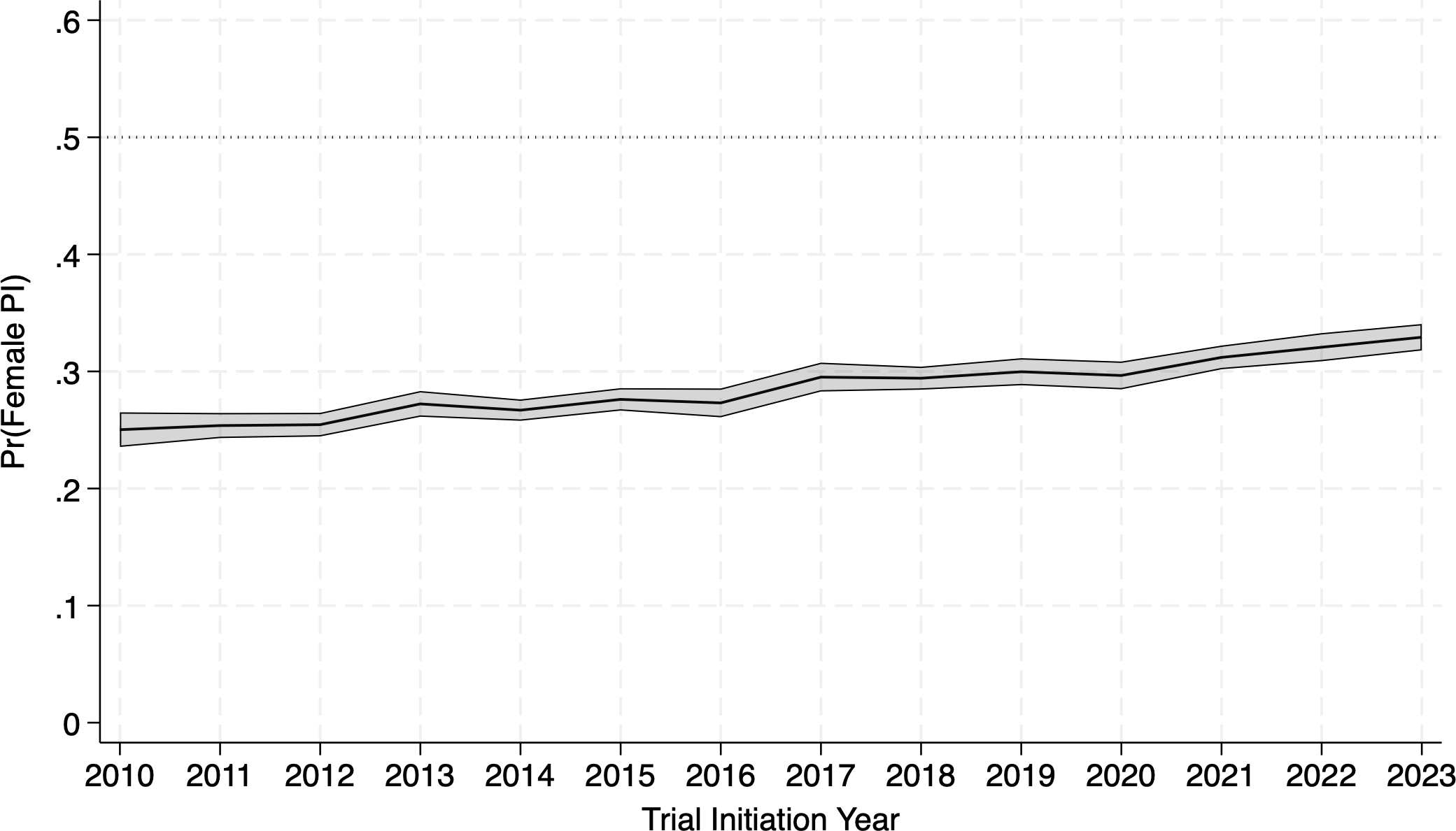


1. Poisson IRR Estimates with Threshold = 0.9.


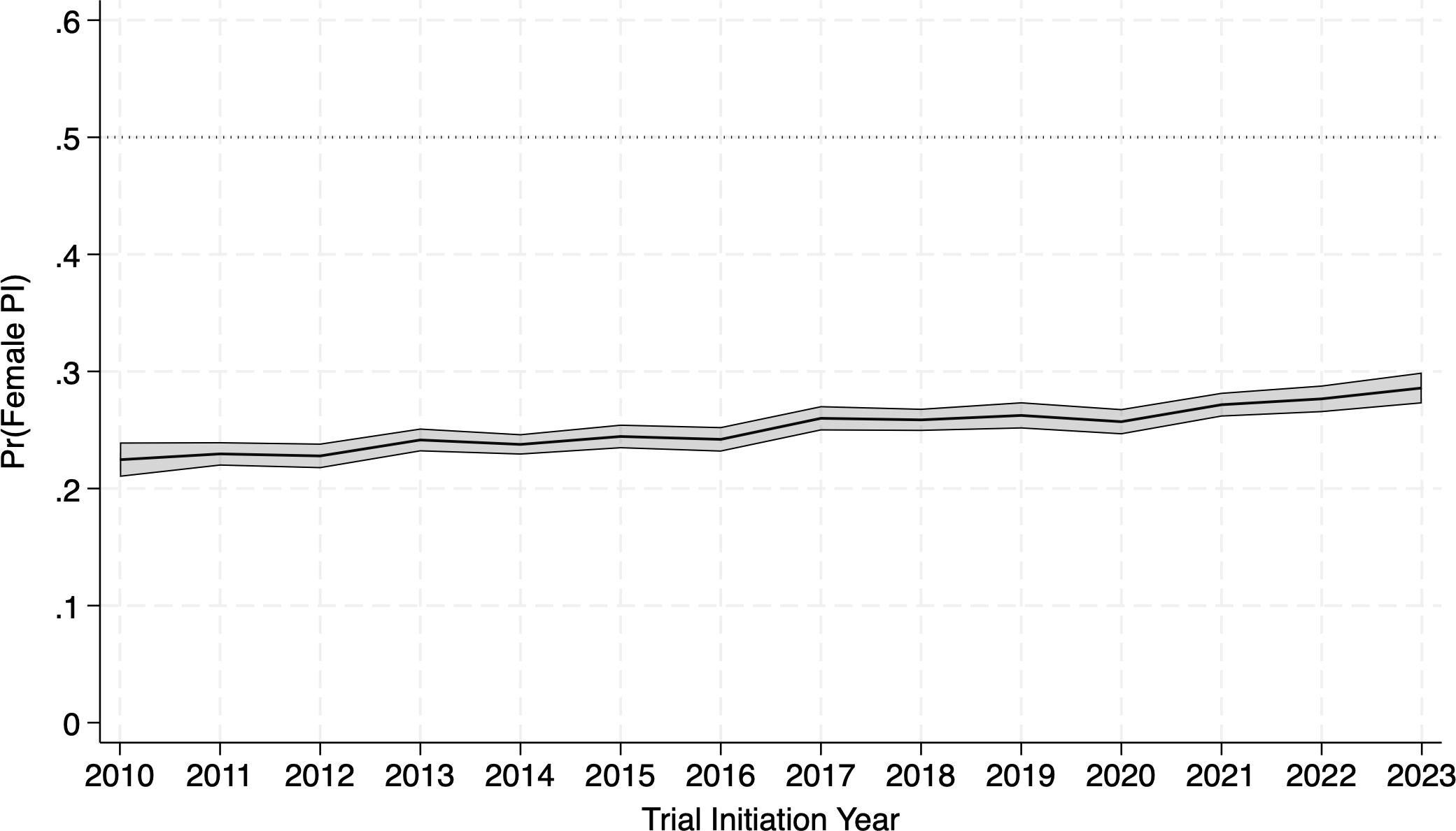


**Figure S.4. Robustness of Gender Differences in Early-Stage Career Trajectories to Alternative Female-PI Classification Thresholds.** This figure reproduces the estimates in [Figure 4](#_bookmark28)b, but classifies principal investigators (PIs) as female using higher probability thresholds of 0.7 (Panel a) and 0.9 (Panel b). All other estimation procedures are as in the main analysis (threshold = 0.5).

1. Poisson IRR Estimates with Threshold = 0.7.


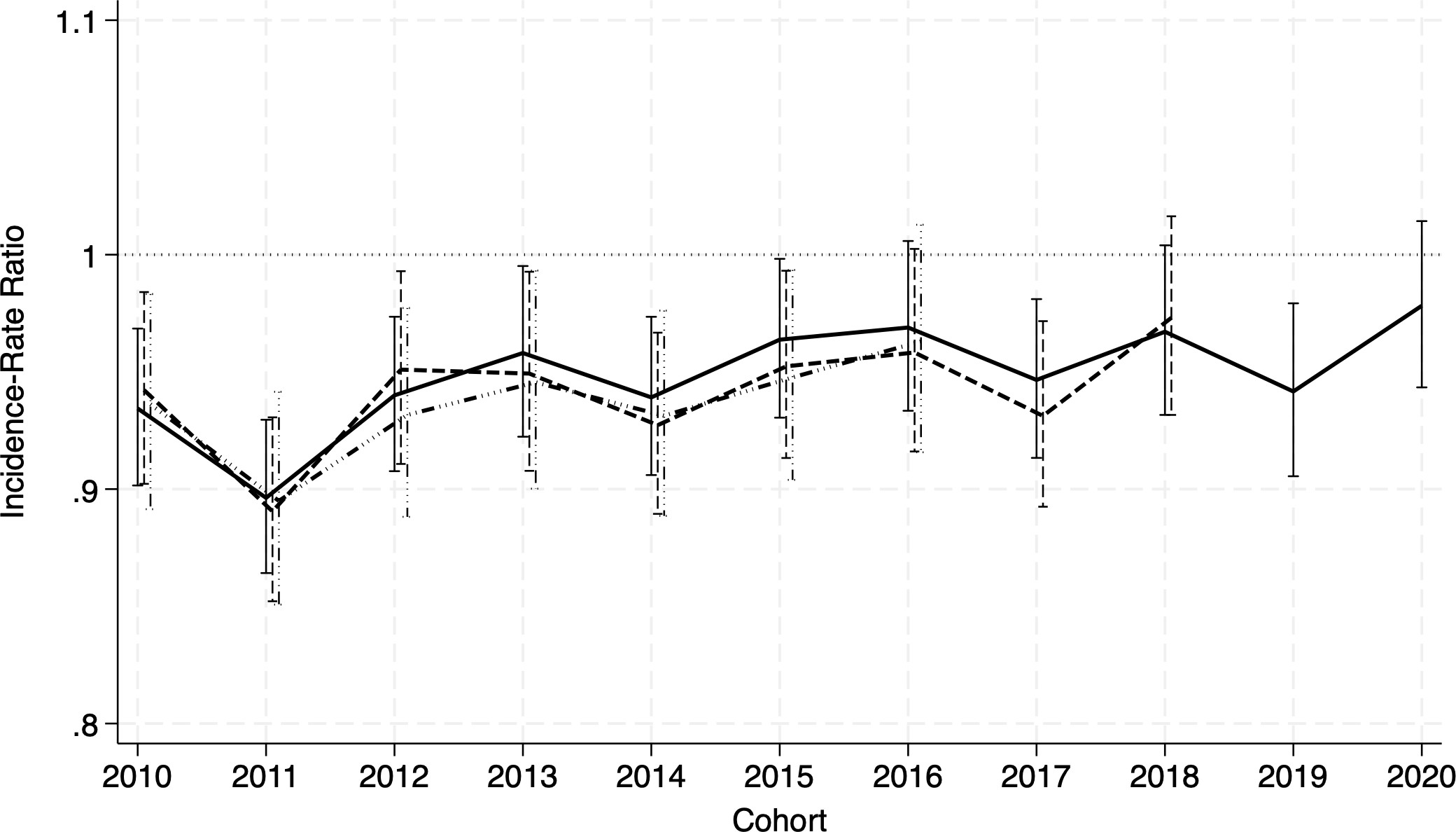


1. Poisson IRR Estimates with Threshold = 0.9.


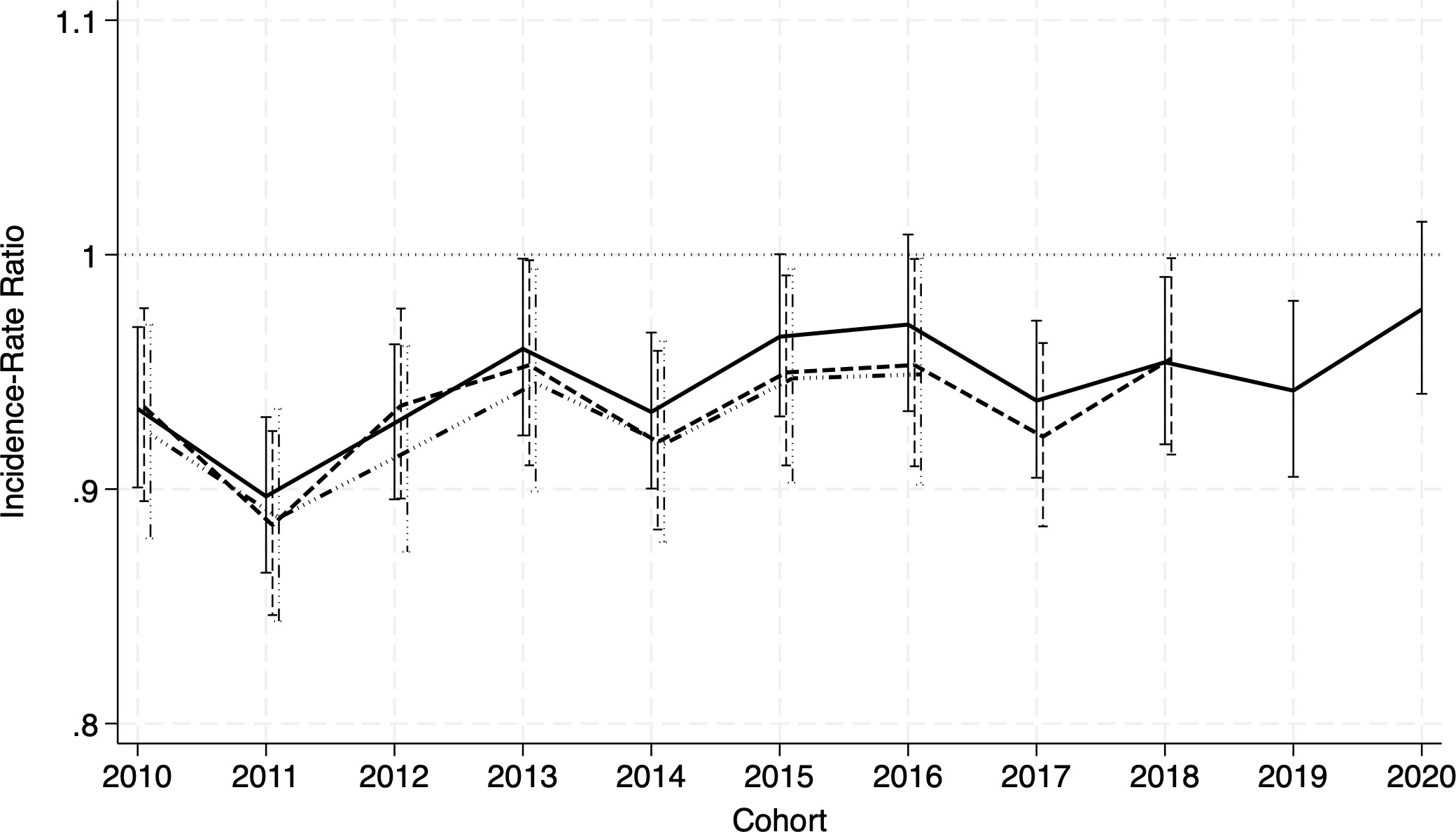


# B Disambiguation of Researcher Affiliation

In the ACCT data, affiliations are often reported inconsistently, with variations in spelling, inclusion of departments, and different suffixes for companies and institutions. To address these challenges, I disambiguate the affiliations. The main steps involved in this process are outlined below:

1. Removal or standardization of special characters, or language-specific characters
2. Removal of Departmental Information: Affiliations that included specific departments were standardized to retain only the main university or research institute name. This approach helps to aggregate data correctly under the main institutional affiliation. Example: “department of obstetrics and gynecology, sohag university hospital, sohag university” was converted to “sohag university”.
3. Handling Pharmaceutical Companies: I removed suffixes such as "inc", "llc", "ltd", "corp", and country-specific identifiers like "us", "usa", "uk" to treat different branches of the same company uniformly. This step ensures that the different branches of a company are correctly aggregated. Example: "janssen research & development, llc" was converted to "janssen pharmaceutical".
4. Preserving Specific Research Institutes: Care was taken to preserve specific research institutes and not to over-generalize them. This step ensures that well-known research entities remain distinct. Example: "institute of cancer research, united kingdom" was retained as "institute of cancer research, united kingdom".
5. Correction of Misspellings: Obvious misspellings were corrected to ensure an accurate representation of affiliations. This helps in further reducing the number of unique but erroneous entries. Example: "northern state medical univercity" was corrected to "northern state medical university".
6. Other examples of Cleaned Affiliations:
   - "department of anesthesiology and pain medicine, seoul national university hospital" converted to "seoul national university"
   - "astellas pharma us, inc." converted to "astellas pharma"
   - "tkl research, inc." converted to "tkl research"
   - "department of family medicine, university of calgary" converted to "university of calgary"
   - "great ormond street hospital for children nhs trust" converted to "great ormond street hospital for children nhs" After applying these disambiguation steps, the number of unique affiliations went from 114,766 to 97,194
